# Vacuolar-ATPase inhibitors are antimicrobial agents active against intracellular mycobacteria

**DOI:** 10.1128/aac.00478-25

**Published:** 2025-10-31

**Authors:** Leah Rankine-Wilson, Tirosh Shapira, Jake Felker, Matthew Christofferson, Henok Sahile, Joseph Chao, Houria Afshar, Raymond J. Andersen, Jim Sun, Yossef Av-Gay

**Affiliations:** 1 Department of Microbiology and Immunology, Life Sciences Institute, University of British Columbia 8166, Vancouver, British Columbia, Canada; 2 Department of Medicine, University of British Columbia 8166, Vancouver, British Columbia, Canada; 3 Department of Chemistry, University of British Columbia 8166, Vancouver, British Columbia, Canada; City St George's, University of London, London, United Kingdom

**Keywords:** *Mycobacterium tuberculosis*, v-ATPase, host-directed therapy, bafilomycin, acidification, PtpA

## Abstract

*Mycobacterium tuberculosis* (Mtb) evades host defenses by inhibiting phagosome acidification in part through the secreted phosphatase PtpA, binding to the vacuolar ATPase (v-ATPase), and disrupting downstream cellular events. We investigated the antimicrobial effects of three v-ATPase inhibitors, Bafilomycin A1 (BafA1), Bafilomycin D, and Cladoniamide B (ClaB), on the growth of Mtb, *M. abscessus* (Mabs), and *M. bovis* BCG in THP-1 and murine infection models. We found potent inhibition of intracellular growth with MIC_50_ in the nanomolar range, with compounds showing a bacteriostatic inhibition of Mtb growth in THP-1 macrophages. Axenic bacteria were not affected by 2 µM compound in broth, although lysate from macrophages incubated with ClaB resulted in a 50% reduction in bacterial growth in broth, which was further enhanced by the addition of zinc. We further discovered that BafA1 amplifies the cytotoxic effects of Mtb infection and limits Mtb’s ability to delay apoptosis in host cells. BafA1 antimicrobial activity was abolished in Mtb PtpA knockout mutant, and BafA1 binding to PtpA was shown via an *in vitro* thermal shift assay. Our findings reveal a complex interplay between v-ATPase inhibition, host cell responses, and bacterial survival, challenging traditional views on phagosome acidification in pathogenesis and suggesting novel avenues for host-directed therapies against intracellular mycobacterial infections.

## INTRODUCTION

Despite significant medical progress, infectious diseases remain a major source of morbidity and mortality worldwide, with antimicrobial chemotherapy being the principal treatment option. The increasing prevalence of antimicrobial resistance poses a significant challenge to the efficacy of conventional antibiotics. Tuberculosis (TB), caused by the obligate human pathogen *Mycobacterium tuberculosis* (Mtb), exemplifies this issue. The current TB treatment regimen is failing on a global scale (1), with patient non-compliance attributed to the regimen’s length, complexity, and toxic side effects. The widespread emergence of antibiotic-resistant TB (2, 3) underscores the urgent need for new treatments to improve patient outcomes. Rapid advancements in biological research have been used to elucidate the complex mechanisms utilized by intracellular pathogens, enhancing novel drug development targets and approaches (4).

The innate immune system plays a pivotal role in bacterial clearance and is particularly influential with respect to intracellular bacterial pathogens that are hidden from humoral defenses. Successful phagocytosis involves a coordinated series of host signaling events that guide early phagosomes into late phagolysosomes, a process broadly characterized by two distinct stages (5–7). The first step involves the sequential decoration of the phagosome membrane with proteins and vacuolar-type H+-ATPase (v-ATPase) pumps, which facilitate vesicle docking and lumen acidification to a pH of ~5.5. This is followed by phagosome-lysosome fusion, where lysosomes deposit lytic cargo into the late phagosome, enabling enzymatic degradation and reducing the phagolysosome pH to below 4.5. Specialized intracellular bacteria, such as Mtb, have developed strategies to avoid phagosome-lysosome fusion and reside in the early phagosome, such as by expressing and secreting the bacterial tyrosine phosphatase, PtpA (8, 9). Although Mtb primarily occupies a modified phagosomal compartment, cytosolic escape can occur and may influence immune recognition, bacterial dissemination, and be responsible for pleiotropic effects of host directed therapies (HDTs) that target intracellular localization (10, 11). The current paradigm dictates that phagosome acidification is deleterious to engulfed bacteria and must be negated by pathogens to survive. However, use of the v-ATPase inhibitor Bafilomycin A1 (BafA1) (12, 13) in the context of intracellular bacterial infection fails to report the increase in infectivity (14–18), as the paradigm would suggest, and reports state that acidification is required for bacterial pathogenicity (19–21). Moreover, in a recent systematic review we conducted (22), and during screening campaigns, we observed antimicrobial activity of BafA1 and other v-ATPase inhibitors against intracellular Mtb.

The macrolide-based BafA1, a potent and specific chemical inhibitor of v-ATPase function, is widely used to study vesicle acidification in autophagy, phagocytosis, and oncology research (23, 24). BafA1 and Bafilomycin D (BafD) belong to the plecomacrolide family of molecules, originally isolated from *Streptomyces griseus* (25). Cladoniamide B (ClaB), (26–28), on the other hand, is a bis-indolylmaleimide alkaloid derived from *Streptomyces uncialis*. The antiviral effects of v-ATPase inhibitors, particularly BafA1, have been demonstrated against SARS-CoV-2 (29–31), rhinovirus (32), and ebolavirus (33). Their suggested mechanism of inhibition primarily involves disrupting essential viral processes such as protease activity, which are not equally pertinent in bacterial infection. Inhibition in bacterial pathogens, including *Legionella pneumophila* (17), *Helicobacter pylori* (34), and *Listeria monocytogenes* (35), has also been shown. Studies using *Salmonella* have reported that BafA1 treatment increases intracellular burden defined by CFU/mL (36), whereas others have shown an inhibitory effect (20, 37, 38). Similarly, conflicting results have been published for *Staphylococcus aureus* (39, 40). Time of addition studies revealed the importance of treatment before the maturation of an established infection, which seems to be a consistent divergence between the conflicting literature. Indeed, reports on the conflicting nature of BafA1 inhibition of Mtb growth have been observed when BafA1 was added after infection was established, negating the inhibitory effect (11).

**Fig 1:**
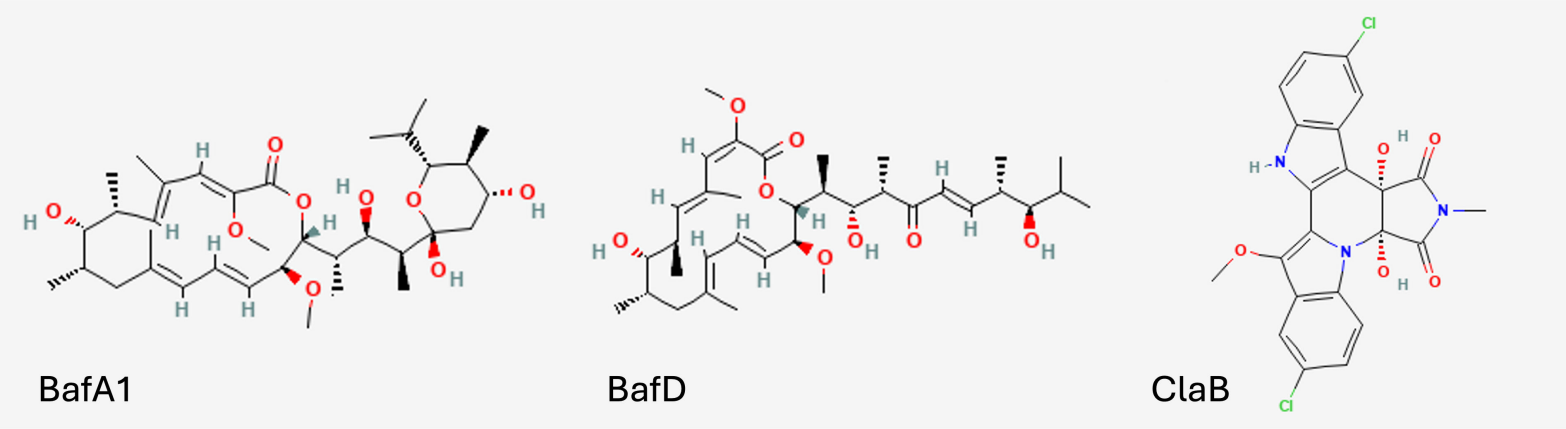
Molecular structures of v-ATPase inhibitor compounds used in this study. Structures obtained from Pubchem.com.

Nevertheless, a comparative study on v-ATPase inhibitors against mycobacterial pathogens has not been performed. We sought to shed light on the antimicrobial profiles of these compounds by investigating the intracellular antimicrobial activity of the v-ATPase inhibitors BafA1, BafD, and ClaB (Fig. 1), against Mtb and *Mycobacterium abscessus* (Mabs) in THP-1 macrophages at sub-cytotoxic concentrations. We found that BafA1 binds to PtpA and amplifies the toxic effect of Mtb intracellular infection, whereas ClaB possesses prodrug-like qualities that result in reduced heavy metal tolerance in bacteria. These findings expand our understanding of the relationship between intracellular pathogenesis and acidification, potentially opening new avenues for therapeutic interventions against intracellular bacterial pathogens.

## RESULTS

### v-ATPase inhibitors Cladoniamide B, Bafilomycin A1, and Bafilomycin D are potent inhibitors of intracellular mycobacterial growth

**Fig 2:**
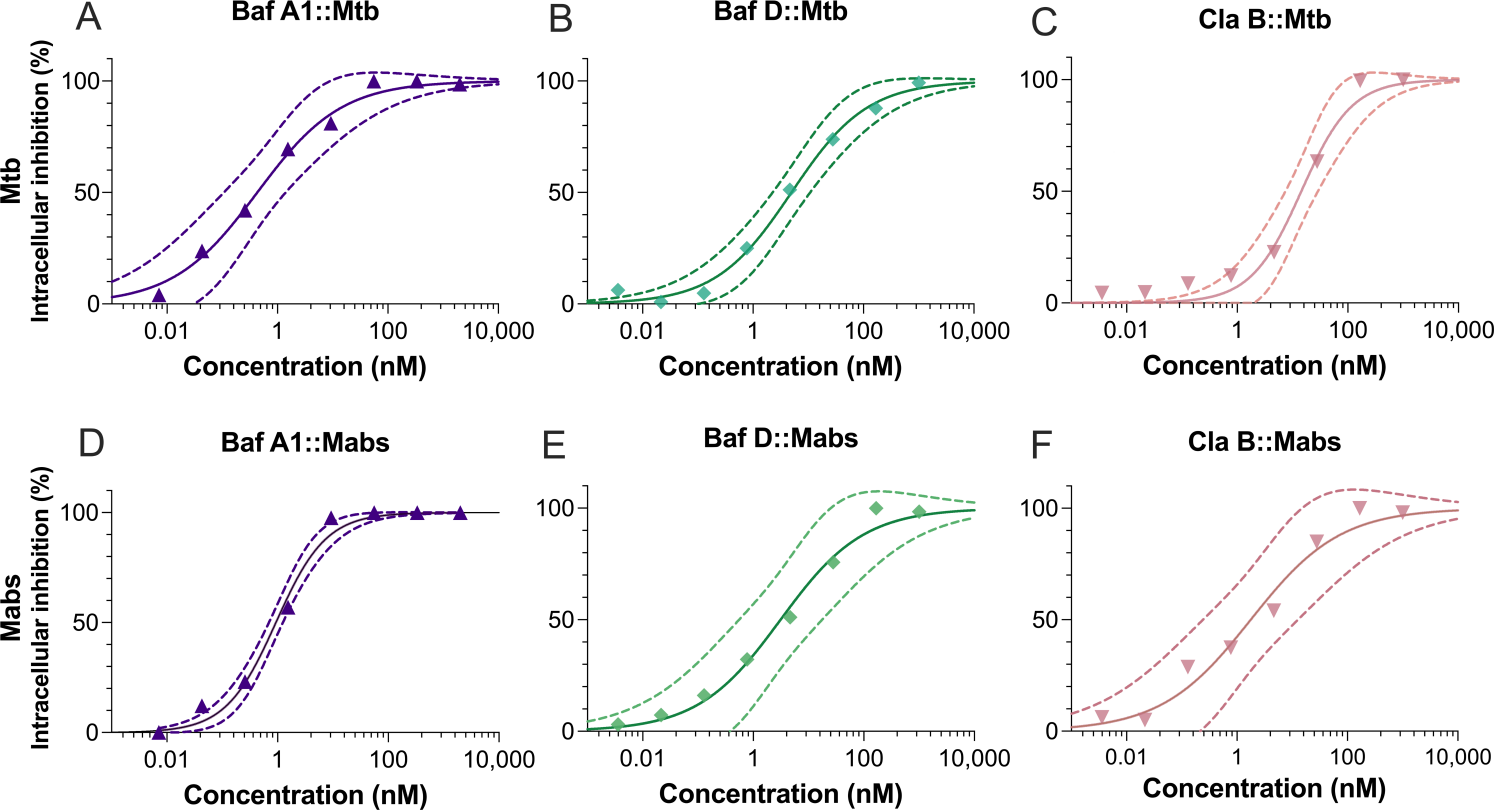
Intracellular growth inhibition by v-ATPase inhibitors in a THP-1 infection model for Mtb and Mabs. BafA1 (purple triangles, **A, D**), BafD (green diamonds, **B, E**), and ClaB (pink inverted triangles, **C, F**) against *Mycobacterium tuberculosis* mc^2^6206 *ΔpanCD ΔleuCD* (Mtb ∆PL) (Mtb, **A, B, C**), and *Mycobacterium abscessus* (Mabs, **D, E, F**). THP-1 cells were infected with MOI 2 (Mtb ∆PL) or 5 (Mabs) for 3 h before extracellular bacteria were washed. Infections were then challenged with the compound for 72 h, and intracellular bacteria were measured by total fluorescent area. Mycobacteria were normalized to 0.1% DMSO (0% inhibition) and 5 µM BDQ (100% inhibition). *n* = 3. Data reported using GraphPad Prism v 10.5.0 using the regression curve: log(inhibitor) vs. response—variable slope (four parameters), least squares fit analysis. Data points indicate mean ± standard error of the mean (SEM) of at least three technical replicates per concentration with biological *n* = 3.

The intracellular activities of the v-ATPase inhibitors BafA1, BafD, and ClaB were tested against Mtb ∆PanLeu auxotroph strain mc^2^ 6206 (Mtb ∆PL) and Mabs and were determined to act in a concentration-dependent manner (Fig. 2). All compounds displayed potent antimicrobial activity with nanomolar MIC_50_ across all treatments with BafA1 challenge of Mtb resulting in an MIC_50_ of 0.431 nM (Fig. 2; Table 1). The highest MIC_50_ was 14.2 nM, observed with Mtb challenged with ClaB. We examined the cytotoxicity of BafA1, BafD, and ClaB against mammalian cells using the MTT assay in THP-1 and HEK293T cells. Compounds showed cytotoxicity at higher concentrations compared with the antimicrobial activity (Table 1). ClaB was the least tolerated of the compounds in THP-1 macrophages with an IC_50_ of 87.52 nM (Table 1). Selectivity indexes (SI) were calculated to compare MIC_50_ and IC_50_ ratios and were all >40, except THP-1:Mtb:ClaB with a SI of 6.1 (Table 1). HEK293T cells showed higher BafD and ClaB tolerability than THP-1 cells, particularly to ClaB, which had an IC_50_ of 620.8 in HEK293T cells (Table 1). BafA1 was active against *M. bovis* BCG at 5 nM, whereas BafD and ClaB were less active even at 20 nM concentration (Fig. S1A). BafA1 was highly toxic to J774 and RAW 246.7 murine-derived macrophage lines, whereas BafD and ClaB were better tolerated (Table 1). Even still, an average of 60.15% (± 9.64) inhibition of Mtb ∆PL growth was observed using 1 nM BafA1 in J774 cells, with lesser inhibition observed for BafD and ClaB (Fig. S1B).

**TABLE 1:** IC_50_ values and selectivity indices of BafA1, BafD, and ClaB against bacterial and mammalian cell lines^*a*^

Cell line	IC_50_ (nM) or selectivity index of:		
	BafA1	BafD	ClaB
IC_50_ (nm)			
THP-1	386.4 (343.7 to 435.2)	481.0 (446.3 to 519.0)	87.52 (76.56 to 100.1)
HEK293T	172.2 (137.9 to 215.4)	869.2 (854.8 to 883.8)	620.8 (596.1 to 646.9)
J774	9.4 (7.05 to 12.27)	513.0 (420.8 to 630.5)	190.1 (147.4 to 248.6)
RAW 264.7	15.7 (12.25 to 20.10)	826.0 (627.0 to 1155)	134.0 (109.3 to 166.0)
Mtb ∆PL	0.431 (0.125 to 1.34)	4.968 (2.34 to 10.60)	14.42 (7.480 to 26.83)
Mtb H37Rv	5.79 (2.20 to 16.49)	N/A	N/A
Mabs	0.933 (0.693 to 1.24)	3.116 (0.6258 to 14.30)	1.774 (0.2788 to 9.863)
Selectivity index (SI): THP-1			
Mtb ∆PL	896.9	96.8	6.1
Mabs	414.2	154.4	49.3
Selectivity index (SI): HEK293T			
Mtb ∆PL	399.7	175.0	43.1
Mabs	184.6	278.9	349.9

^*a*^ The IC_50_ values (nM) are presented with 95% confidence intervals in parentheses for THP-1 human macrophages, RAW 264.7 and J774 murine macrophages, HEK293T epithelial cells, *Mycobacterium tuberculosis* ∆PanLeu auxotroph strain (Mtb ∆PL), Mtb H37Rv BSL3 virulent strain (Mtb H37Rv), and *Mycobacterium abscessus* ATCC 19,977T rough form (Mabs). Selectivity indices (SI) were calculated as the ratio of mammalian cell (THP-1 or HEK293T) IC_50_ to bacterial IC_50_, with higher values indicating better selective toxicity against bacteria versus host cells. MTT assay and bacterial inhibition were measured 48–72 h post-challenge. Data reported as mean ± standard error of the mean (SEM) from at least three technical replicates with *n* = 3-5. Non-linear regression was performed on GraphPad Prism for Mac V. 10.3.0 using the [Inhibitor] vs. response (three parameter) tool. NA: No confident inhibition identified at 20 nM.

### v-ATPase inhibitors induce unique host cell death phenotypes in cells during active infection

Controlling the intracellular growth of Mtb by modulation of macrophage apoptosis was recently shown as an attractive drug target for HDT (5) and drug repurposing campaigns (41). v-ATPase inhibitors were shown to induce apoptosis due to a breakdown of the autophagy pathway associated with their inhibition of vesicle acidification (23, 42). Indeed, a decrease in host-cell survival was observed even at low concentrations in preliminary infection experiments. To test if BafA1 treatment preferentially kills infected cells, we compared an alternative measure of host cell survival via whole cell counts of infected versus non-infected macrophages and found a greater infection-dependent loss of THP-1, with a 50% detachment of cells at 222 nM of non-infected THP-1 cells compared with 47 nM for Mtb (∆PL) infected cells (Fig. S2A). Mtb inhibition in the infected cells was distinct from THP-1 death determined by this in-plate comparison experimental set. We next measured nuclear condensation, a key morphological feature of cells undergoing apoptosis (43), and found a moderate relationship between v-ATPase concentration and apoptosis in uninfected cells after 48 h of treatment. There was no significant difference in apoptosis between each compound (Fig. S2B).

**Fig 3:**
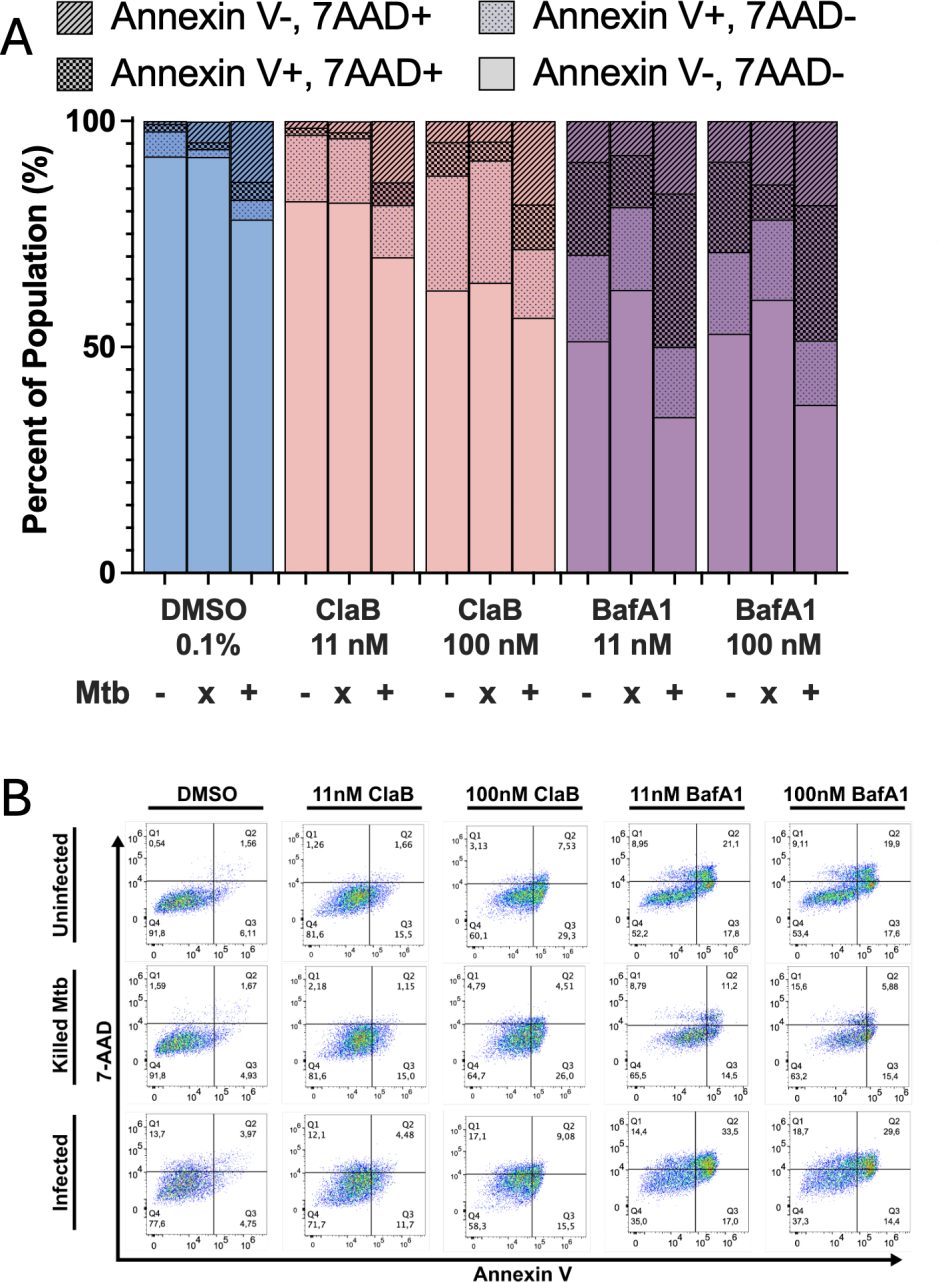
Mtb infection exacerbates bafilomycin-induced host cell death in THP-1 macrophages. THP-1 macrophages were infected with either live (+) or killed (x) *Mycobacterium tuberculosis* mc^2^6206 *ΔpanCD ΔleuCD* (Mtb ∆PL), followed by treatment with Bafilomycin A1 (purple) or Cladoniamide B (pink) (11 or 100 nM) or DMSO vehicle control (blue) for 48 h. Uninfected macrophage treatments are noted as (-). Mtb was killed by incubating the bacteria in 5 µM of bedaquiline for 2 h. Cell viability, apoptosis, and necrosis were assessed using Annexin V/7-AAD staining. Live cells were negative for both 7-AAD and Annexin V (no pattern), early apoptotic cells were positive for Annexin V only (dot pattern), cells with 7-AAD and Annexin V-positive staining were grouped as necrotic and late apoptotic (checkered pattern), whereas necrotic cells were positive for 7-AAD only (diagonal line pattern). (**A**) Bar plots summarize the percentage of each death mode for the treatment condition. (**B**) Representative flow cytometry plots display cell populations across each treatment condition, categorized into live, early apoptotic, late apoptotic/necrotic, and necrotic cells. Data reported from at least three biological and technical replicates.

Based on these findings, we continued our investigations with BafA1 and ClaB as representative plecomacrolide and bis-indolylmaleimide v-ATPase inhibitors, respectively. To delineate the specific mode of cell death activated by v-ATPase inhibition, we performed Annexin V and 7-AAD staining to distinguish between cells undergoing early/late apoptosis and necrosis (44). We observed that the process of particle phagocytosis, as demonstrated by infection with 4 µm polystyrene bead particles, did not exacerbate the cell death induced by BafA1 or ClaB (Fig. S3A). Mtb is known to control host cell death processes (5, 45), and we indeed saw that infection with live Mtb (∆PL) significantly increased the percentage of late-stage dead and double-stained cells but not apoptotic cells (Annexin V+) compared with DMSO controls. This effect was not observed when macrophages were infected with killed Mtb, indicating that active infection is required to trigger necrotic responses. Similarly, infection with live, but not antibiotic-killed Mtb, did exacerbate the inhibitor-induced cell death, suggesting that BafA1 either triggers or amplifies the cytotoxic effects of active Mtb infection (Fig. 3). Importantly, the cell death caused by live Mtb infection is distinct from that observed from live Mtb treated with inhibitor (Fig. S3B and C). Further analysis revealed that bafilomycin-induced cell death during Mtb infection triggered a late apoptotic/necrotic mode of cell death as indicated by increased proportions of Annexin V and 7-AAD double-positive cells, whereas early apoptotic events (single Annexin V+) remained relatively stable compared with non-infected conditions (Fig. 3). ClaB treatment, however, resulted in increased necrosis-associated staining. Interestingly, ClaB treatment resulted in retention of the healthy-cell population compared with BafA1, suggesting that it possesses an alternative mechanism of antibacterial activity.

**Fig 4:**
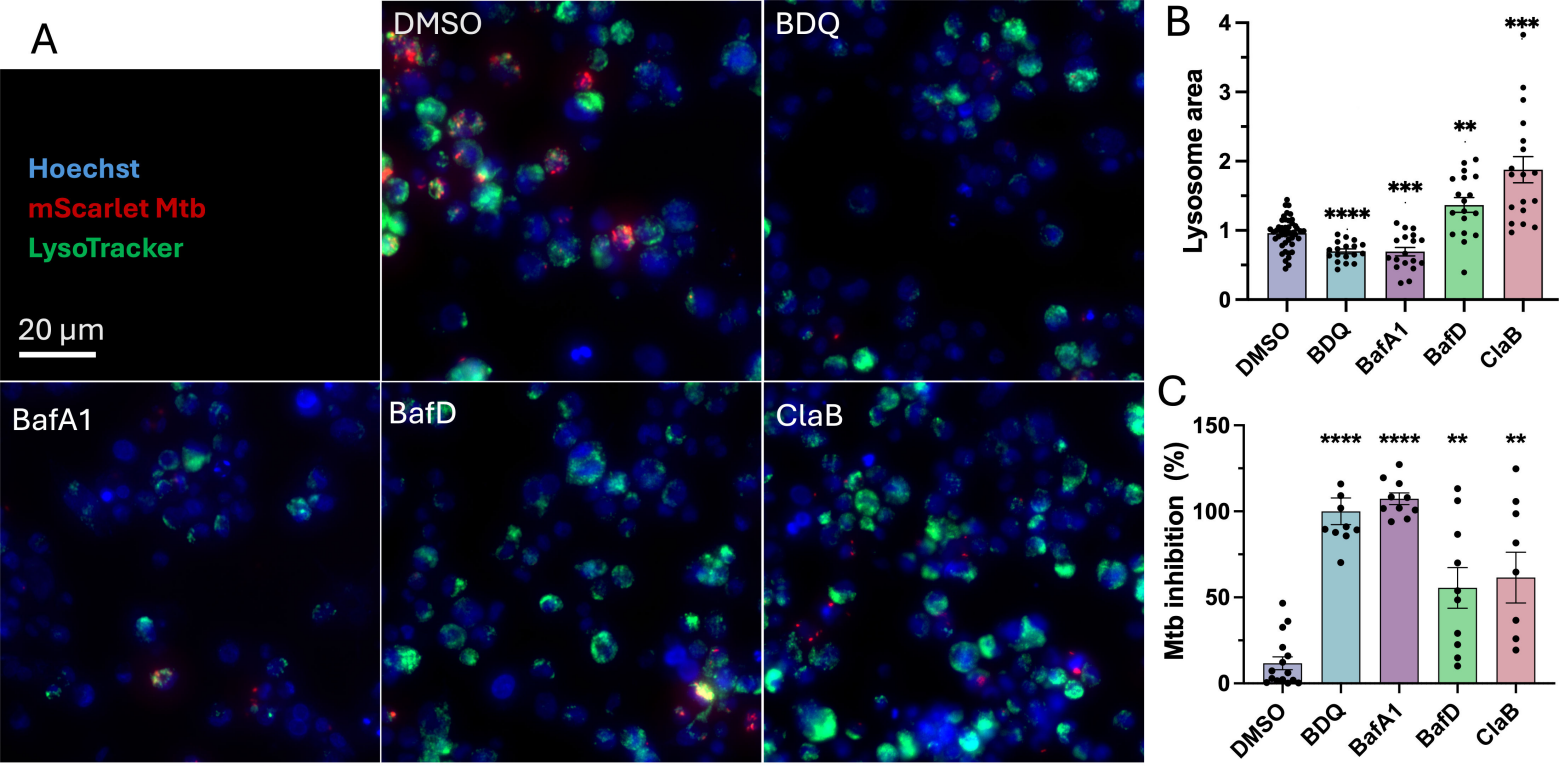
Vesicle acidification after 72 h v-ATPase inhibitor challenge during Mtb infection: Representative micrographs (**A**) and accompanying lysosome area (**B**) and Mtb inhibition (**C**) data of THP-1 cells infected with an MOI of 2 *Mycobacterium tuberculosis* mc^2^6206 *ΔpanCD ΔleuCD* (Mtb ∆PL) harboring integrative mScarlet fluorescence (red) and treated with either v-ATPase inhibitors Bafilomycin A1 (BafA1, 5 nM), Bafilomycin D (BafD, 20 nM), and Cladoniamide B (ClaB, 20 nM), or control compounds of bedaquiline (BDQ 5 µM) and DMSO (0.1%) for 72 h. The cells were washed thrice to remove cell debris and extracellular bacteria and then stained with 1 µg/mL Hoechst 33342 nuclear stain (blue) and 100 nM LysoTracker Green DND-26 (green) in dPBS for 30 min at 37°C. The staining solution was removed for fresh, warm dPBS for immediate imaging on CX5 using a 20× objective colocalization protocol. To account for background staining, lysosomes were identified as bright objects of > 2 SD from channel mean. Bar graphs indicate the total fluorescence area of LysoTracker normalized to the mean total LysoTracker area of DMSO wells per plate. BafA1 returned 0.69-fold less lysosomes than ± 0.26 SD, *P* < 0.0001. BafD 1.37-fold lysosome area (± 0.45 SD, *P* < 0.005) and ClaB 1.88-fold (± 0.80 SD, *P* < 0.005). Mtb inhibition in these experiments was reported as 100% (± 10.7 SD, *P* < 0.0001) for 5 nM BafA1, 55.53% inhibition (± 37.45 SD, *P* < 0.005) for 20 nM BafD, and 61.53% (± 44.23 SD, *P* < 0.005) for 20 nM ClaB. Data reported as mean ± standard error of the mean (SEM) of at least three biological and technical replicates. Statistics indicate significance from one-way ANOVA with Dunnett’s post-test compared with DMSO. *P < 0.05, **P < 0.01, ***P < 0.001, ****P < 0.0001.

To monitor the effects of acidification over the course of infection, we followed acidification throughout the 72 h of compound challenge at single concentrations. Using LysoTracker staining to identify vesicles that fused with lysosomes, lysosome area was normalized to 0.1% DMSO (Fig. 4A and B); 5 nM BafA1 sustained reduced acidification and lysosomal fusion after 72 h compared with DMSO, whereas 20 nM BafD and 20 nM ClaB showed over 2-fold greater acidification, respectively. In these experiments, Mtb (∆PL) growth was again strongly inhibited with 5 nM BafA1 (100% inhibition), whereas 20 nM each BafD and ClaB inhibited less, at 55.53% and 61.53%, respectively (Fig. 4C).

### Cladoniamide B, Bafilomycin A1, and Bafilomycin D are bacteriostatic against intracellular bacteria

**Fig 5:**
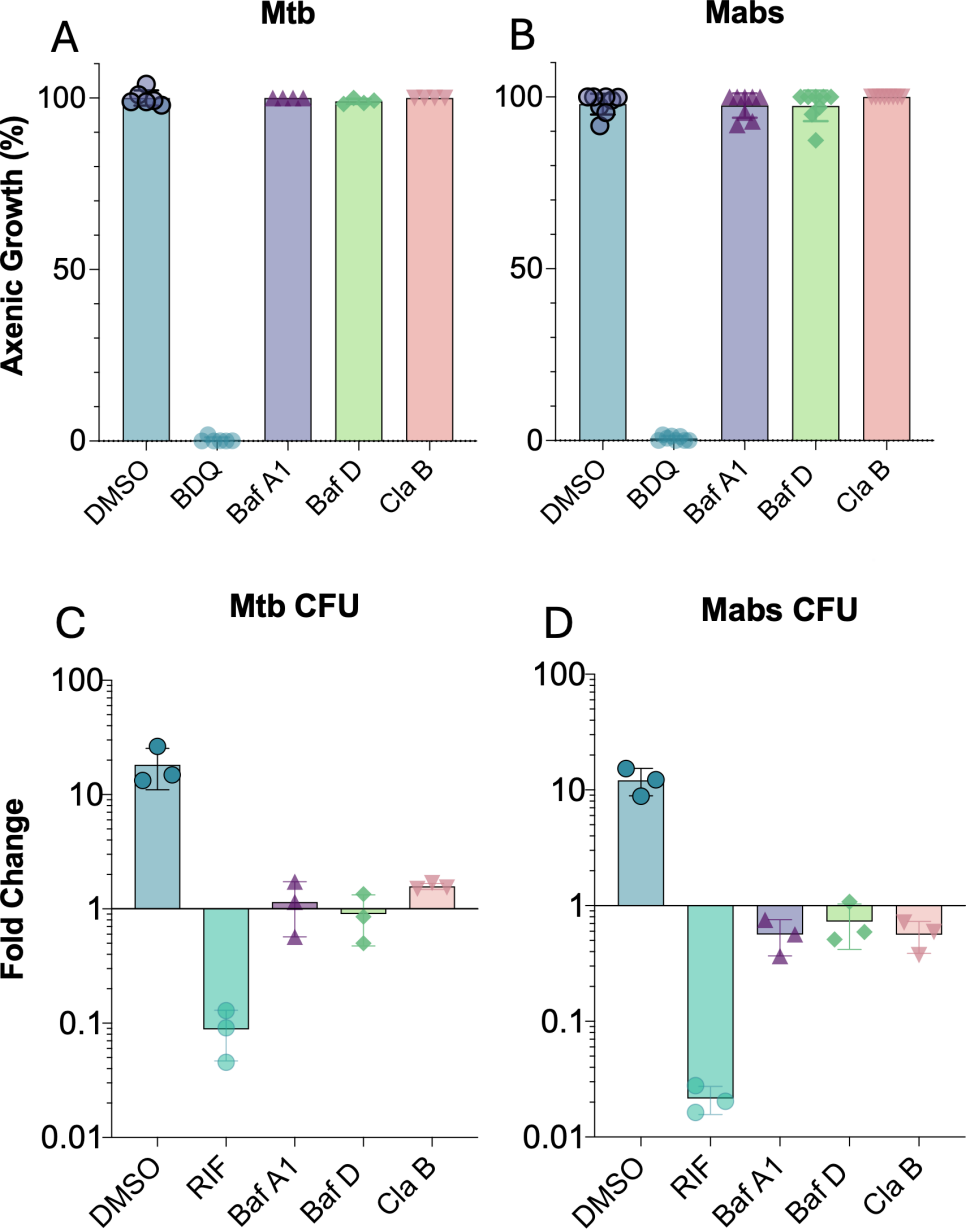
Effects of v-ATPase inhibitors on bacterial growth. (**A and B**) Axenic growth of bacteria with 2 µM v-ATPase inhibitors Bafilomycin A1 (purple), Bafilomycin D (green), and Cladoniamide B (pink), in 7H9 (*Mtb* ∆PL, **A**) and (Mabs, **B**) with appropriate supplements. Mtb and Mabs grew for 7 days and 4 days, respectively, and growth was measured using OD_600_. Data normalized to 0.1% DMSO (blue, 0% inhibition), and 5 µM bedaquiline (turquoise, BDQ: 100% inhibition). (**C and D**) CFU counts of THP-1 macrophages treated with 10 nM v-ATPase inhibitors and infected with Mtb (**C**) or Mabs (**D**). Counts were transformed to fold change from matched infection counts in CFU/mL from immediately after infection (T0), (Tfinal/T0). Colonies were grown on 7H10 agar with appropriate supplements for mycobacteria. Controls were bactericidal antibiotics rifampicin (5 µM, RIF). Data reported as mean ± standard error of the mean (SEM) of at least three technical replicates and are representative of three biological repeats. Accompanying data can be found in the supplemental material.

Bafilomycins show potent antifungal activity yet possess negligible direct antibacterial activity (25). We confirmed the lack of direct antibacterial properties of BafA1, BafD, and ClaB against Mtb and Mabs at concentrations up to 2 µM in broth (Fig. 5A and B). As the antibacterial effects of the inhibitors are limited to intracellular growth, we next sought to determine if these compounds are bacteriostatic or bacteriolytic in THP-1 macrophages by counting colony-forming units (CFU) after drug challenge compared with immediately after infection (T_0_). The bactericidal agent rifampicin (46) at 5 µM was used as a positive control for bactericidal agents in mycobacteria. Compared with T_0_, BafA1, BafD, and ClaB treatments had a limited effect on CFU, suggesting a bacteriostatic mechanism of inhibition (Fig. 5C and D).

**Fig 6:**
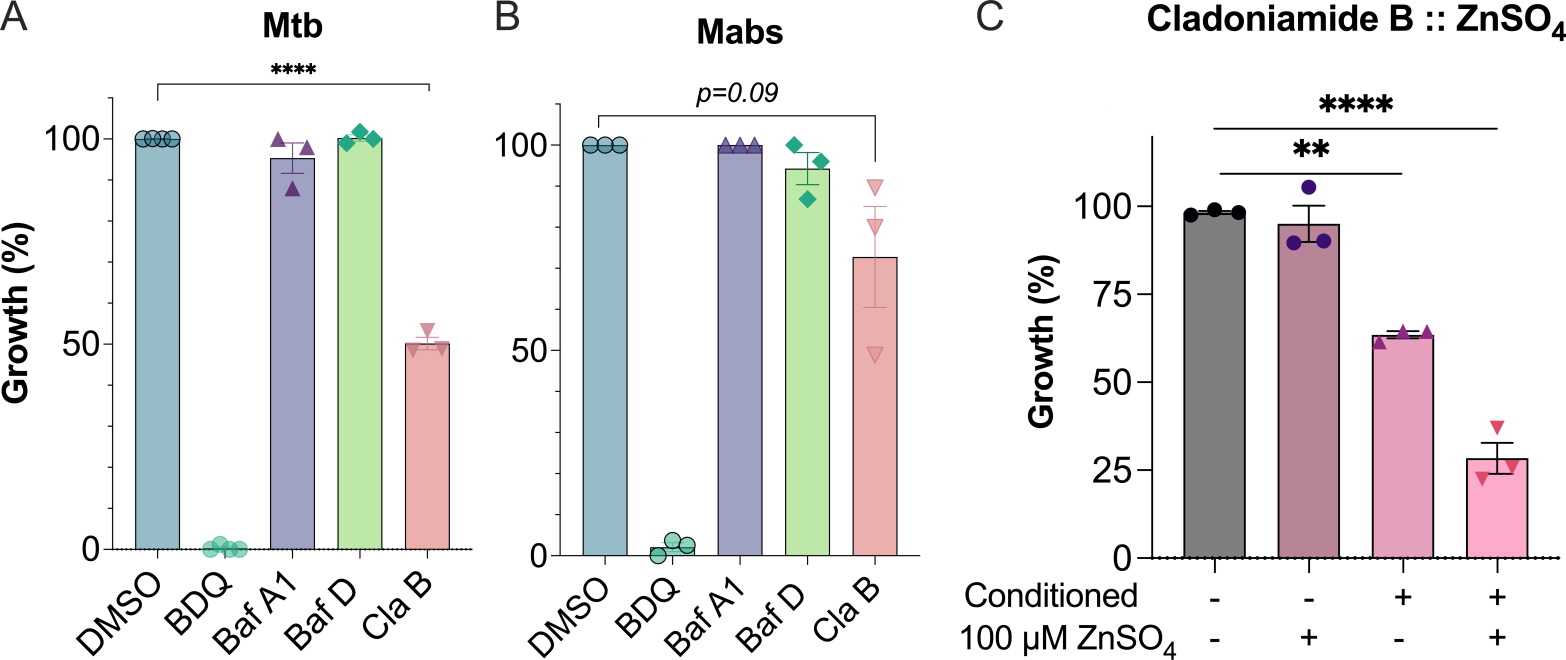
Cladoniamide B conditioned lysate media inhibits planktonic Mtb, and its direct antimicrobial activity is enhanced in the presence of zinc. (**A**-**B**) Planktonic growth of bacteria in v-ATPase inhibitor:THP-1 conditioned lysate media. Bafilomycin A1 (purple), Bafilomycin D (green), and Cladoniamide B (pink). Mtb (Mtb mc^2^6206 *ΔpanCD ΔleuCD* (Mtb ∆PL) and Mabs were grown for 7–14 days and 4 days, respectively, and growth was measured using OD_600_. Data normalized to 0.1% DMSO conditioned media (100% growth) and 5 µM bedaquiline (BDQ), conditioned media representing 0% growth. (**C**) Inhibition of Mtb axenic growth in ClaB-conditioned lysate media is enhanced with the addition of zinc. Mtb were grown in fresh (conditioned -) or conditioned (conditioned +) ClaB media in the presence or absence of 100 µM ZnSO4 for 7–14 days. Growth was normalized to 0.1% DMSO (100% growth) or 5 µM BDQ (0% growth) in respective fresh or conditioned RPMI1640. Data reported as mean ± standard error of the mean (SEM) of at least three biological and technical replicates. Statistics indicate significance from one-way ANOVA with Dunnett’s post-test compared with DMSO. *P < 0.05, **P < 0.01, ***P < 0.001, ****P < 0.0001.

We tested whether the v-ATPase inhibitors possess pro-drug-like activities by incubating the compounds with THP-1 cells, then exposing bacteria to the macrophage extract containing the drug, termed “conditioned lysate.” Limited by the cytotoxicity of the compounds to the macrophages, we used 100 nM of the compounds. BafA1 and BafD from macrophage extracts did not inhibit any bacterial growth (Fig. 6A and B. In contrast, Mtb (∆PL) but not Mabs growth was inhibited in the presence of ClaB conditioned media, at 50.3% and 26.8% inhibition, respectively. This suggests that ClaB is modified within macrophages and that metabolites of ClaB could have a direct bacterial target. To further evaluate this, the conditioned lysate from ClaB-treated macrophages was analyzed at different time points using LC-MS and observed a reduction of ClaB UV peak intensity over a period of time (Fig. S4), suggesting possible metabolism.

ClaB was shown to inhibit the yeast zinc transporter encoded by *zhf1* (28). To test whether ClaB or its metabolites act against metal ion transport in Mtb, we challenged ClaB and ClaB:THP-1 conditioned media with 100 µM ZnSO_4_ and assessed growth via OD_600_ over 14 days. ClaB did not inhibit Mtb growth in the presence or absence of zinc (Fig. 6C), whereas conditioned ClaB caused an average of 34% reduction in Mtb (∆PL) growth. Adding zinc to ClaB-conditioned lysate resulted in a 74.2% reduction of growth compared with control.

### BafA1 concentration-dependent antimicrobial phenotype is abolished during THP-1 infection with Mtb ∆PtpA

**Fig 7:**
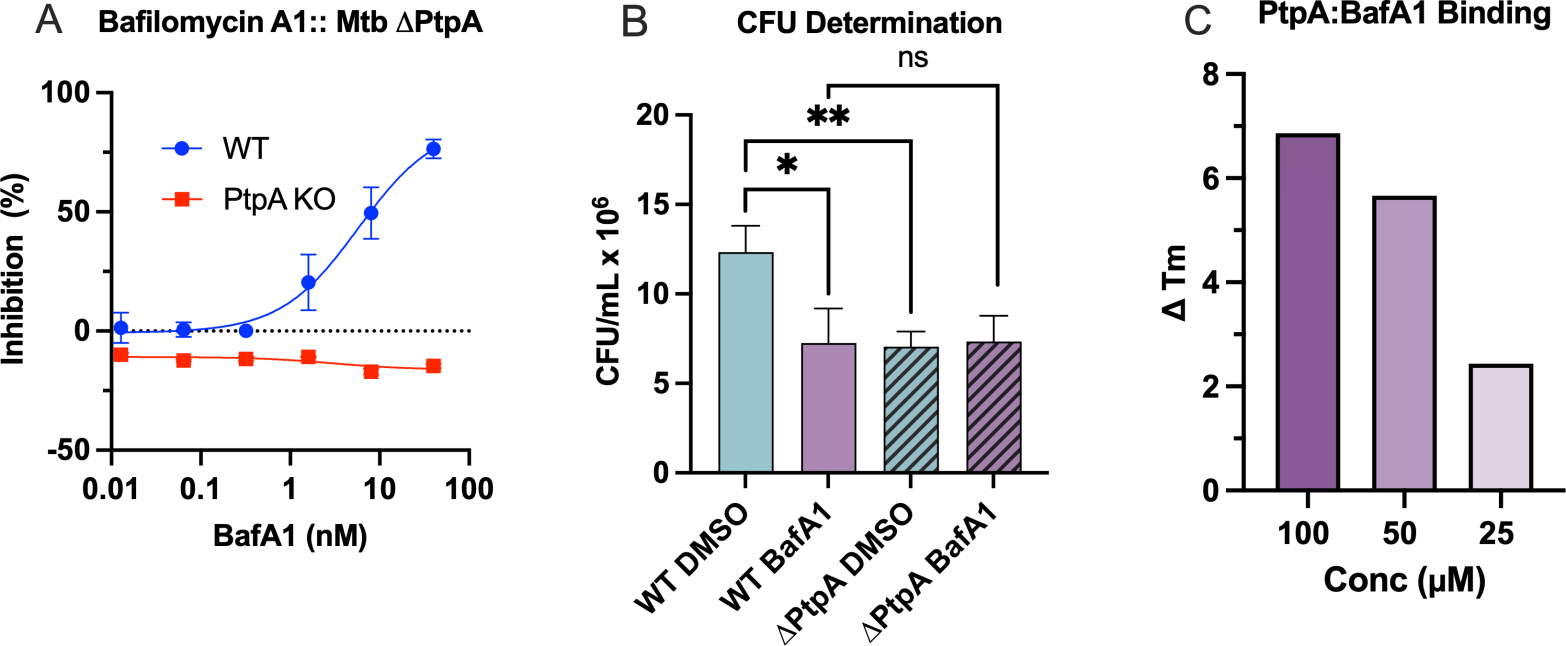
Bafilomycin A1 binds to PtpA, contributing to the intracellular antimicrobial activity being abrogated in the Mtb PtpA deletion mutant. (**A**) High-content analysis of intracellular growth of BSL3 Mtb H37Rv WT and ∆*ptpA*. Intracellular Mtb were measured 72 h post-infection by fluorescent area and normalized to 0.1% DMSO (0% inhibition) and 5 µM BDQ (100% inhibition) for each respective strain. Negative values indicate growth above the DMSO control as a normalized proportion to BDQ and DMSO controls. Non-linear regression was performed for WT on GraphPad Prism for Mac V. 10.3.0 using the [Inhibitor] vs. response (three parameter) tool. Data reported as mean ± standard error of the mean (SEM) of at least three technical replicates with *n* = 3. In-plate quality control was performed using Z’ factors, which were calculated for each strain independently to be each > 0.4. (**B**) CFU determination: CFU counts of THP-1 macrophages treated with 5 nM Bafilomycin A1 and infected with Mtb H37Rv WT or ∆PtpA mutant for 72 h. Colonies were grown on 7H10 agar with 10% OADC. DMSO control 0.1% vol/vol. Data reported as mean ± standard error of the mean (SEM) of at least three technical and biological replicates. Statistics indicate significance from one-way ANOVA with Dunnett‘s post-test compared with DMSO. *P < 0.05, **P < 0.01, ***P < 0.001, ****P < 0.0001. (**C**) Bafilomycin A1 resulted in a concentration-dependent positive shift in the melting temperature (ΔTm) of PtpA *in vitro*, as measured by differential scanning fluorimetry (DSF).

As v-ATPase function is actively inhibited by Mtb protein tyrosine phosphatase PtpA, we used an Mtb H37Rv ∆*ptpA* knockout to investigate v-ATPase inhibitor activity compared with the parental wild-type (WT) Mtb strain. No confident inhibition was observed in BafD or ClaB treatments of 20 nM in WT. Strikingly, BafA1 treatments from 0.1 to 50 nM did not correlate with a reduction in Mtb ∆*ptpA* growth compared with DMSO control (Fig. 7A), although the virulent Mtb H37Rv strain reported an average MIC_50_ of 5.79 nM under the BSL3 conditions tested. High content results for BafA1 were confirmed by CFU assay (Fig. 7B; Fig. S5) with growth of WT Mtb treated with 5 nM BafA1 not statistically different from ∆*ptpA* vehicle control. This led to the hypothesis that BafA1 could directly interact with PtpA or its substrates. We used a Differential Scanning Fluorimetry (DSF) assay to assess the binding of BafA1 to purified Mtb-PtpA. DSF measures ligand-induced thermal stabilization by detecting shifts in the melting temperature (ΔTm) of PtpA across a thermal gradient (25–80°C), where a positive ΔTm is indicative of direct target engagement (47). BafA1 stabilized PtpA in a dose-dependent manner, resulting in a ΔTm of 6.9 at 100 µM (Fig. 7C; Table 2).

**TABLE 2:** Bafilomycin A1 binds to PtpA: thermal shift assay^*a*^

	DMSO 1%	BafA1 100 µM	BafA1 50 µM	BafA1 25 µM
v_50_ (°C)	39.85	46.71	45.51	42.29
95% CI	N/A^*b*^	37.16 to 50.54	0 to 50.66	0 to 47.10
*P*		<0.0001	<0.0001	<0.0001

^*a*^ Statistics indicate significance from one-way ANOVA with Dunnett‘s post-test compared with DMSO. *P < 0.05, **P < 0.01, ***P < 0.001, ****P < 0.0001.

^*b*^ N/A, data not reliably calculable.

## DISCUSSION

This study details the potent bacteriostatic activity of v-ATPase inhibitor compounds against intracellular mycobacterial pathogens. We show that the activity of BafA1 is limited to intracellular infection associated with chemical inhibition of v-ATPase activity, which induces host cell death in infected macrophages. With further scrutiny, we found that ClaB possesses pro-drug qualities, and its direct activity is enhanced with the addition of zinc. Finally, we showed that deletion of PtpA abolishes BafA1 antimicrobial activity in THP-1 macrophages, leading to the demonstration that BafA1 directly binds to the Mtb secreted protein PtpA.

By evaluating the effects of v-ATPase inhibitors on the survival of intracellular pathogens, we provide evidence to support the argument that a canonical understanding of acidification in the scope of bacterial pathogenesis and host defense is an oversimplified dogma (48). Phagosome acidification plays a dual role in bacterial survival within host cells (49–51); complete attenuation of phagosome acidification can disrupt pH-induced regulatory mechanisms, potentially impairing bacterial survival, while enhanced phagosome acidification can also limit bacterial viability, such as in the case of imatinib, which has been shown to inhibit intracellular Mtb (52). Although there is clinical evidence to suggest that imatinib treatment may increase susceptibility to Mtb infection (53). The data imply that Mtb relies on a finely tuned phagosomal environment for optimal survival and that perturbing this balance, either by excessive acidification or by blocking acidification altogether, impairs bacterial fitness. The dynamic relationship between acidification and inhibition is further evidenced by the sustained inhibition of lysosomal acidification by BafA1 over 72 h, being closely linked to its suppression of intracellular Mtb growth. This sustained effect of BafA1 suggests that impairing acidification, either by inhibitors or by PtpA, may serve as a prelude to signal for blocking phagosome-lysosome fusion. BafD and ClaB allow partial recovery of acidification and are less effective. As ClaB and BafD retain some antimicrobial activity despite partial acidification recovery, it suggests that lysosomal pH modulation is independent of their direct antimicrobial effects. Understanding these interactions is crucial for developing targeted therapies that can effectively combat intracellular pathogens without inadvertently aiding their survival.

Cladoniamides are indolotryptoline drugs and act on v-ATPases as well as the yeast zinc transporter Zhf1. Disruption of zinc regulation may also underlie ClaB toxicity to THP-1 cells. This suggests that BafA1 and BafD act primarily through host v-ATPase-inhibitory or host-directed activities, although we cannot rule out intracellular modification that cannot be detected within the limitations of our extraction assay. Although preliminary LC-MS analysis shows metabolism of ClaB by the host, further investigation of ClaB and its analytes' antimicrobial properties was constrained by limited compound availability and cellular toxicity. The retention of a healthy cell population with ClaB treatment compared with BafA1 further supports a differential mechanism of inhibition as a result of direct antibacterial activity. Synthesizing and testing a small library of possible metabolites is a valid strategy for uncovering active prodrugs, as demonstrated by Rybniker and colleagues (54); therefore, a comprehensive metal toxicology panel and structure-activity relationship analysis of possible analytes would address remaining questions and is recommended for future studies.

Similarly, a full analysis of synergy between v-ATPase inhibitors and front-line antimicrobial agents would better define their potential role as adjunctive therapies, as BafA1 treatment has been shown to limit the front-line TB drug pyrazinamide (PZA) due to PZA activity being enhanced in acidic environments (11). The BafA1 and ClaB antibacterial activity against Mtb is more potent than its cytotoxic effects on host cells, suggesting an appropriate therapeutic window exists. As v-ATPases lack tissue specificity and vesicular acidification is an integral function to cellular homeostasis, from a therapeutic perspective, treating TB patients with v-ATPase inhibitors is unlikely to be feasible due to these safety concerns. Indeed, we showed that the variability of toxicity is greatly dependent on cell line, with murine macrophages almost completely intolerant of BafA1 treatment, particularly during infection. A further limitation to consider is the extent to which the host-directed mechanism of v-ATPase inhibitor-induced bacterial inhibition affects bacteria located outside the vacuole, given that Mtb can also persist in damaged vacuoles or within the cytosol (10, 11). Additional high-resolution microscopy studies, particularly those incorporating immunostaining, would help clarify the relationship between vacuolar localization and the efficacy of inhibition in this context. However, the study remains scientifically valuable as it underscores the importance of understanding how host cell death pathways can be manipulated to control intracellular pathogens like Mtb.

Neutralizing acidic compartments enables researchers to visualize and study pH-dependent cellular processes such as autophagy, endocytosis, and protein trafficking. These processes are critical for understanding cellular mechanisms that play essential roles in diseases like cancer and neurodegenerative disorders (55). Indeed, some studies utilized BafA1 strictly as a biochemical tool to report *Salmonella* infection is controlled by autophagy without considering bafilomycin’s associated intracellular antimicrobial effects (36). The discovery that BafA1’s antimicrobial activity depends on PtpA means that BafA1 should be cautiously considered a probe of vesicular acidification in infection models. As its effects are context-dependent, the use of bafilomycin as an autophagy-related tool should be limited to growth-independent infection studies, and careful consideration should be paid to any associated conclusions, particularly in relation to Mtb. We have shown that BafA1 exerts its intracellular activity in part by promoting cytotoxicity in the infected macrophage, producing late apoptotic and necrotic cells containing mycobacterial cargo. This can potentially be utilized for a new concept utilizing apoptosis and cell death as targets for host-directed therapy, as recently shown (5).

Mtb effector phosphatase PtpA directly inhibits assembly and function of v-ATPase on the phagosome surface (8, 9). Our group has previously shown that Mtb H37Rv ∆*ptpA* mutants’ intracellular survival is significantly attenuated, further highlighting the importance of phagosome acidification in successful phagocytic processing and infection control. Nevertheless, when ∆*ptpA* was challenged with BafA1, we saw no concentration-response inhibition of intracellular bacterial growth compared with WT. BafA1-treated ∆*ptpA*, BafA1-treated WT, and DMSO-treated ∆*ptpA* all display similar CFU counts. This suggests that BafA1’s antimicrobial activity against intracellular Mtb includes a specific interplay with PtpA or its downstream effects. Thus, the findings highlight that both lysosomal acidification and host cell toxicity are central to the effects of v-ATPase inhibitors and that PtpA’s broad impact on host cell processes is critical for understanding the context and limitations of BafA1’s antimicrobial activity. This calls for further studies to delineate the direct and indirect contributions of acidification, toxicity, and effector-mediated host modulation in Mtb pathogenesis and therapy. Considering the difference in MIC_50_ between Mtb ∆PL and H37Rv, strain-to-strain variation is possible and worth further exploration. Disrupting vesicular acidification could target professional intracellular pathogens that depend on acidic environments for optimal growth or the activation of critical virulence factors (48, 49). The initial and moderate phagosome acidification driven by v-ATPases serves as a cue to regulate the bacteria’s intracellular response, including the activation of niche-specific genes essential for progression of pathogenicity. This hypothesis is strengthened by the loss of inhibitory effect seen if BafA1 is challenged 24 h post-infection (11, 19). Together, this suggests inhibiting vesicular acidification could exploit the delicate pH balance that many intracellular pathogens depend on to sense and adapt to their intracellular niche, ultimately leading to attenuation within host cells.

In conclusion, this study sheds light on the complex interplay between v-ATPase inhibitors, intracellular bacterial pathogens, and host cell responses, challenging our understanding of phagosome acidification in bacterial pathogenesis and opening new avenues for potential therapeutic interventions against intracellular infections. Although the potent *in vitro* antimicrobial activity of these compounds is promising, further research is needed to fully elucidate their mechanisms of action and evaluate their potential as novel direct (ClaB) or host-directed (BafA1, BafD) therapies for combating intracellular bacterial infections and promote further examination of BafA1 in TB drug discovery.

## MATERIALS AND METHODS

### Compounds

Bafilomycin A1 (13038-1) and Bafilomycin D (19438) were purchased from Cayman Chemical (MI, United States). Cladoniamide B was kindly supplied by Raymond Andersen (UBC, Canada). Bedaquiline (TMC-207) was purchased from AddoQ (CA, United States). All compounds were solubilized in DMSO.

### Bacterial strains and culturing

Virulent *Mycobacterium tuberculosis* strain H37Rv (dTomato pTEC27), H37Rv ∆PtpA mutant (dTomato pTEC27), BSL2-safe *Mycobacterium tuberculosis* mc^2^6206 *ΔpanCD ΔleuCD* (Mtb ∆PL) (56, 57), *Mycobacterium bovis* Bacillus Calmette-Guérin (BCG) (dTomato pTEC27), and *Mycobacterium abscessus* ATCC 19,977T, R (rough form, integrative mScarlet vector) (58) were routinely grown at 37°C in standing cultures in 7H9 broth (Difco Middlebrook) supplemented with 10% (vol/vol) OADC (5% bovine albumin fraction, 2% dextrose, 0.004% catalase, 0.05% oleic acid, and 0.8% sodium chloride solution), 0.05% (vol/vol) Tween-80 (Sigma-Aldrich), noted as 7H9-OADC-T throughout. mc^2^6206 media was supplemented with 50 µg/mL L-leucine and 24 µg/mL pantothenate. Strains harboring mScarlet integrative fluorescence were grown with 50 mg/L kanamycin, and pTEC27 dTomato strains were grown with 50 mg/L hygromycin.

### Infection of THP-1 macrophages

THP-1 monocytes were cultured as previously described (59). For assays using Mtb mc^2^6206 auxotrophic strains, incomplete RPMI 1640 medium was supplemented with 50 µg/mL L-leucine and 24 µg/mL pantothenate. At least 24 h before infection, THP-1 monocytes were differentiated into macrophage-like states using phorbol 12-myristate 13-acetate (PMA; 40 ng/mL).

### Mycobacterial infection

Bacteria were washed once in 7H9 media containing 0.05% Tween 80. The pellets were then re-suspended in RPMI1640 medium, de-clumped using a 25G blunt syringe, and OD_600_ was measured (Mtb OD_600_ of 1 ≈ 3 × 10^8^ CFU/mL; Mabs OD_600_ of 1 ≈ 1 × 10^9^ CFU/mL) and opsonized with 10% human serum for 30 min at 37°C. Bacteria were added to THP-1 macrophages at a multiplicity of infection (MOI) of 2:1 and incubated for 3 h at 37°C with 5% CO_2_ as previously described (59–61). After infection, wells were washed with RPMI 1640 three times to remove extracellular bacteria, and the compound was added and incubated for 3 days. After incubation, the cells were washed thrice and fixed with 4% paraformaldehyde in PBS for 60 min, the fixative was removed, and stained with 1 µg/mL Hoechst 33342 in PBS.

RAW 246.7 murine-derived macrophages were routinely grown in Dulbecco’s modified Eagle medium (DMEM, Gibco) with 10% FBS and appropriate supplements for bacterial infection. Cells were seeded at 5 × 10^3^ cells per well in 96-well plates and allowed to adhere overnight before being infected with 10 bacteria per macrophage as per the THP-1 infection protocol above.

### High content analysis parameters

Intracellular bacterial growth was monitored using the CellInsight CX5 High Content Platform (Thermo Fisher Scientific) using methods previously described by our group (58–62). Briefly, macrophages were identified and counted through nuclear staining, and a mask was created to represent the entire cell or region of interest (ROI). Second channel fluorescence (Red:569/593 nm, Green:485/521 nm) was used to detect bacteria “spots” inside the cellular ROI. These spots were quantified using a variety of measurements, including intensity and area. Fluorescent measurements closely correspond with CFU as previously validated (62).

### *In vitro* growth assay

Bacteria were seeded at an OD_600_ of 0.02 in OD_600_ at day 3 for Mabs and day 7 for Mtb. DMSO (0.1%) was used as the 100% growth negative control, and 5 µM BDQ for 0% normalization controls. Normalized values outside the 0% to 100% range were set to their closest range value. OD_600_ was read on a VarioSkan Plate Reader at 600 nm.

### Conditioned lysate assay

Differentiated THP-1 macrophages were challenged with 100 nM of v-ATPase inhibitor, 5 µM BDQ, or equimolar DMSO for 24 h. Following, cells were mechanically lysed and the solution was centrifuged, then aspirated to remove any cellular debris. This “conditioned lysate” was added to 96-well plates at 150 µL per well after being inoculated with 7.5 µL of bacteria from a concentrated stock of OD_600_ = 1. Bacterial growth was measured by OD_600_.

### Compound cytotoxicity

3-(4,5-Dimethylthiazol-2-yl)−2,5-diphenyltetrazolium Bromide (MTT) assay was used to determine cell survival at varying concentrations of drug. Briefly, THP-1 or HEK293T cells were seeded into a 96-well plate at 1 × 10^5^ or 1 × 10^4^ cells per well, respectively, and either allowed to adhere (HEK293T, RAW 264.7, J774) or differentiated overnight (THP-1). Cells were washed and serial dilutions of compounds added, then incubated with 0.1% saponin or 0.1% DMSO controls for 50–60 h, after which 200 µg of MTT was added and cells incubated for a further 2.5 h. MTT/formazan was solubilized by adding 100 µL of extraction buffer (20% wt/vol of SDS dissolved in 50% N, N-dimethylformamide containing 2.5% acetic acid and 2.5% 1 M HCl) and incubated overnight. Plates were read on a VarioSkan Plate Reader at 570 nm.

### Cell death analysis

THP-1 macrophages were infected with green, fluorescent 4 µm polystyrene microspheres (Bangs Laboratories Inc.) or Mtb mc^2^6206 expressing GFP at an MOI of 10:1 for 4 h. Killed Mtb was generated by treatment with 5 µM BDQ for 2 h at 37°C. After infection, macrophages were washed thrice with PBS, then treated with 11, 33, or 100 nM of bafilomycin A1 or DMSO vehicle for 48 h. Following treatment, supernatant and a single PBS wash were collected and pooled. Macrophages were then detached using TrypLE Express (Thermo Fisher Scientific) and combined with the supernatant/wash mixture. Cells were pelleted, washed, and stained with Alexa Fluor 647 Annexin V (BioLegend) for 15 min at room temperature and washed twice with Annexin V binding buffer (10 mM HEPES, 140 mM NaCl, 2.5 mM CaCl_2_). Cells were then stained with 7-AAD viability staining solution (eBioscience) for 15 min at room temperature. Cells were then analyzed via flow cytometry to quantify proportions of live, necrotic, and apoptotic cells using the Cytek Northern Lights system (Cytek Biosciences). Data were analyzed using the SpectroFlo software (Cytek Biosciences) and FlowJo V10 software (BD Biosciences).

### Liquid chromatography mass spectrometry of host cell lysates

HEK293T cells were seeded at a density of 6 × 10^4^ cells/mL overnight, then 5 mL of compounds was added at 600 nM for 1, 5, and 24 h. After the given time, cells were mechanically scraped, collected, and centrifuged at 4,000 × *g* for 10 min. The supernatant was collected and filtered through a 0.2 µm syringe filter. The filtrate was concentrated by lyophilization, redissolved in DMSO/methanol (1:1), and analyzed by LC-MS (Agilent 1260 Infinity II LCMS). LC-MS conditions: Solvent A consisted of water with 0.1% TFA. Solvent B consisted of acetonitrile with 0.1% TFA. The method involved gradient elution as follows: flow 0.5 mL/min, the first 3 min (90% A, 10% B), till 9 min (from 95% A, 10% B to 10% B, 90% A), maintained at this gradient (10% A, 90% B) for another 3 min then from 12.01 to 14 min at 90% B, 10% A. The wavelength for UV detection was 254 nm.

### Lysosome staining and imaging

Differentiated THP-1 cells were infected with Mtb ∆PL as previously described (60). At 72 h post-infection (p.i.), the cells were washed twice with warm Dulbecco’s phosphate-buffered saline and 1 µg/mL Hoechst 33342, 100 nM LysoTracker Green 26 (Thermo Fisher Scientific) in dPBS was added for 30 min at 37°C. Staining solution was removed for fresh, warm dPBS for imaging on CX5 using a 20× objective. To account for background staining, lysosomes were identified as bright objects of >2 SD from channel mean. The lysosome signal area was normalized to the mean of DMSO control wells.

### DSF assay

The DSF experiment was conducted following the procedure described previously (48, 63). In a 96-well plate (Applied Biosystems MicroAmp Fast Optical 96-Well Reaction Plate, 0.1 mL), recombinant PtpA protein was incubated at a final concentration of 2.5  µM in DSF assay buffer (20  mM HEPES, 100  mM NaCl, pH 7.2) with SYPRO Orange at 1× (from a 5000× stock, Sigma Aldrich). BafA solutions were added after SYPRO Orange, bringing the final reaction volume to 25  µL. Reactions were run in technical triplicates on a StepOnePlus Real-Time PCR System (Thermo Fisher Scientific), with a thermal gradient from 25°C to 80°C, and fluorescence readings were taken every 1°C to generate PtpA melt curves.

## SUPPLEMENTAL MATERIAL

Supplemental figures aac.00478-25-s0001.docx Fig. S1 to S4.

Supplemental data aac.00478-25-s0002.xlsx Raw data.

ASM does not own the copyrights to Supplemental Material that may be linked to, or accessed through, an article. The authors have granted ASM a non-exclusive, world-wide license to publish the Supplemental Material files. Please contact the corresponding author directly for reuse.
