# Supplementary material for: Vacuolar-ATPase inhibitors are antimicrobial agents active against intracellular mycobacteria
Source: Antimicrob Agents Chemother. 2025 Oct 31;69(12):e00478-25. doi: 10.1128/aac.00478-25 (PMC12691628; doi:10.1128/aac.00478-25)
Supplement: Supplemental figures — Fig. S1 to S4. [file aac.00478-25-s0001.docx]

Vacuolar-ATPase inhibitors are antimicrobial agents active against intracellular mycobacteria

Supplementary figures.

**
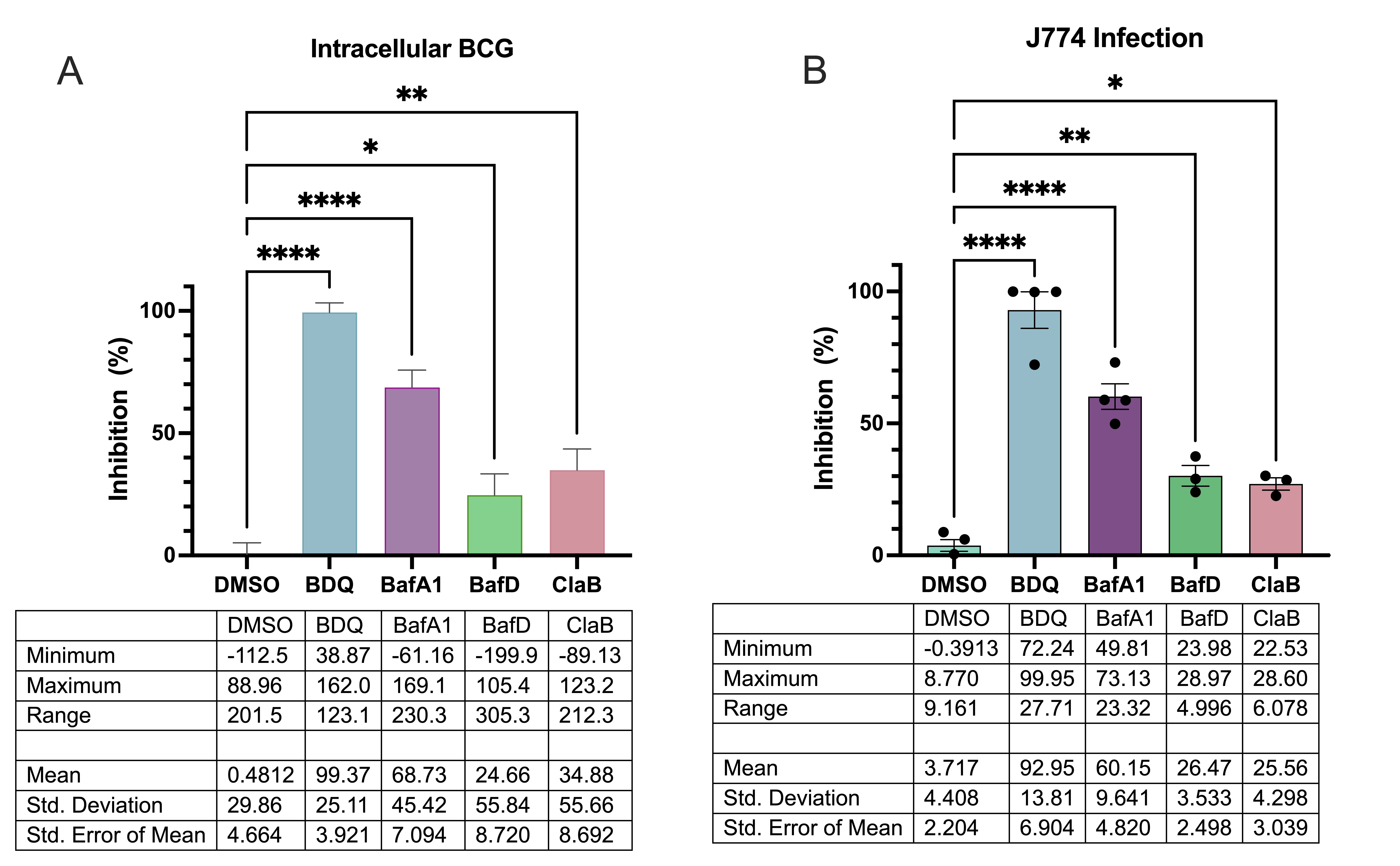
**

**Supplemental Figure 1. Single concentration v-ATPase inhibition of intracellular *Mycobacterium bovis* BCG in THP-1 cells (A) and intracellular Mtb ∆PL in J774 macrophages.**

(A) THP-1 infection of *Mycobacterium bovis* BCG was performed for three hours at an MOI of 10 bacteria per macrophage then challenged with Bafilomycin A1 (BafA1, 5 nM), Bafilomycin D (BafD, 20 nM) and Cladoniamide B (ClaB, 20 nM), including controls of bedaquiline (BDQ 5 μM) and DMSO (0.1%) for 72 hours when intracellular bacteria were enumerated by total fluorescent area. (B) J774 murine macrophages were seeded at a density of 5 x 10^3^ cells per well in 96 well plates the night before infection. Then *Mycobacterium tuberculosis* mc^2^6206 *ΔpanCD ΔleuCD* (Mtb ∆PL) was performed for three hours at an MOI of 2 bacteria per macrophage then challenged with Bafilomycin A1 (BafA1, 1 nM), Bafilomycin D (BafD, 20 nM) and Cladoniamide B (ClaB, 20 nM), including controls of bedaquiline (BDQ 5 μM) and DMSO (0.1%) for 72 hours when intracellular bacteria were enumerated by total fluorescent area.

Results are shown as normalized to DMSO (0% inhibition) and BDQ (100% inhibition). Data reported as mean ± standard error of the mean (SEM) of at least three technical replicates with n=3. Statistics indicate significance from one-way ANOVA with Dunnett ‘s post-test compared to DMSO. * p < 0.05, ** p < 0.01, *** p < 0.001, **** p < 0.0001.

**Supplemental Figure 2. Bafilomycin A1 effect on THP-1 survival measured by whole cell count with and without Mtb ∆PL infection.** A) Hoechst 33342-stained nuclei were enumerated using high-content analysis after 72 hours drug challenge. Cells were washed thrice with media before fixative and stain was added. Cell counts were calculated proportionally to uninfected THP-1 cells with 0.1% DMSO (100% survival). Non-linear regression was performed on GraphPad Prism for Mac V. 10.3.0 using the [Inhibitor] vs. response (three parameter) tool. Data reported as mean ± standard error of the mean (SEM) of at least three technical replicates with n=3. IC50 uninfected was 222nM (95% CI 159-303 nM) versus infected at 47 nM (95% CI 24-88 nM) with two-way ANOVA p < 0.001 for infected vs uninfected cell counts. Two-way ANOVA comparing Mtb inhibition (0.68 nM 90% CI 0.25-1.9 nM) for this matched set of experiments and infected THP-1 cell loss p < 0.0001.

B) THP-1 cells were treated with varying concentrations of v-ATPase inhibitor and apoptosis was roughly measured using the Autoptosis^1^/apooptosis nuclear condensation method. Hoechst 33342-stained nuclei were enumerated using high-content analysis after 48 hours drug challenge. Bafilomycin A1 (purple triangles), Bafilomycin D (green diamonds) and Cladoniamide B (pink). Cells were live imaged and bright, condensed nuclei were counted as apoptotic. Data was normalised to 4 mM H_2_O_2_ (100%) and 0.01% DMSO (0% apoptosis) in-plate controls. Non-linear regression was performed on GraphPad Prism for Mac V. 10.3.0 using the [Agonist] vs. response -- Variable slope (four parameters) tool. Data reported as mean ± standard error of the mean (SEM) of at least three technical replicates with n=3.

^1^ Rens C, Shapira T, Peña-Diaz S, Chao JD, Pfeifer T, Av-Gay Y. Apoptosis assessment in high-content and high-throughput screening assays. Biotechniques. 2021 Jun;70(6):309-318. doi: 10.2144/btn-2020-0164. Epub 2021 Jun 11. PMID: 34114488.

**
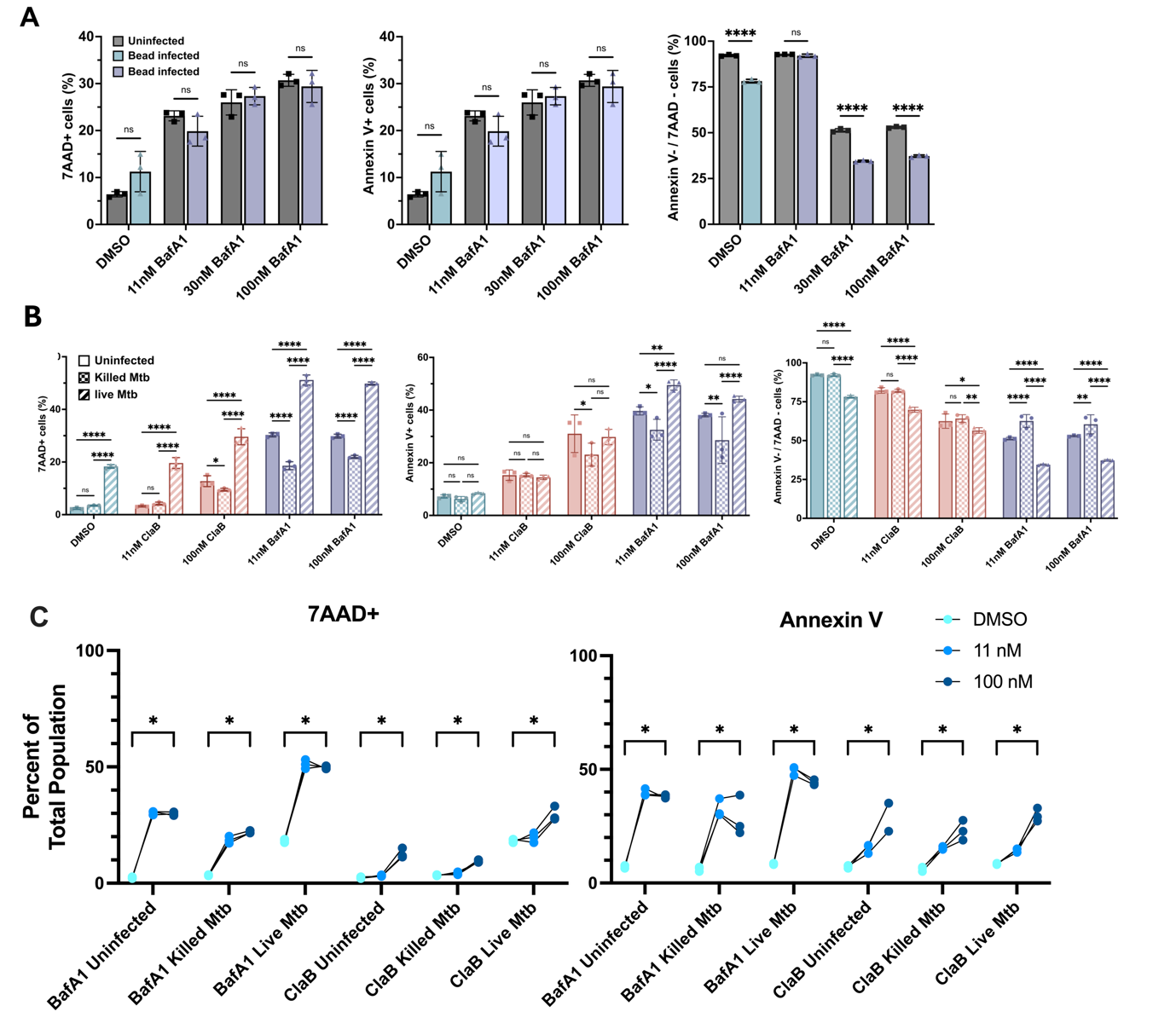
**

**Supplemental Figure 3. Bafilomycin A1 and Mtb induce host cell apoptosis and necrosis in THP-1 macrophages.** THP-1 macrophages were infected with either (A) 4 µm polystyrene microspheres as a non-biological control or (B) Mtb ∆PL, followed by treatment with varying concentrations of bafilomycin A1 (purple), (11, 33, or 100 nM) or Cladoniamide B (pink), (11 nM or 100 nM) or a 0.1% DMSO (blue), vehicle control. Mtb was killed by incubating the bacteria in 5 µM of Bedaquiline for 2 hours. Cell viability, apoptosis, and necrosis were assessed using Annexin V/7-AAD staining. Representative bar plots summarize the percentage of each cell population for each treatment condition. Data shown in this figure are representative of n = 3 replicate experiments. Two-way ANOVA was performed: n.s., non-significant; *, p<0.05; **, p<0.01; ***, p<0.001, ****, p<0.0001. (C) Data from (B) presented as increasing concentrations of compound from DMSO vehicle control (light blue) to 100 nM v-ATPase inhibitor (dark blue). Statistics indicate significance from one-way ANOVA with Dunnett ‘s post-test compared to DMSO, and all p values are < 0.005. Data and statistics for DMSO:11 nM comparisons are provided in the data availability file of this manuscript.

**Supplemental Figure 4: LC-MS run summary showing the metabolism of ClaB overtime in macrophages.** LSMS analysis was done after 1 hr (a), 5 hr (b) and 24 hr (c) incubation of HEK293T cells with 1 μM ClaB. ClaB’s UV peak decreased over time indicating the possible biotransformation (metabolism) by the cells.

**Supplemental Figure 5: Representative colony forming unit determination of Bafilomycin A1 treatment on *Mycobacterium tuberculosis* H37Rv PtpA deletion mutant versus H37Rv wildtype.** CFU counts of THP-1 macrophages treated with 5 nM v-ATPase inhibitor Bafilomycin A1 (purple) or 0.1% DMSO (blue) vehicle control and infected with Mtb H37Rv WT (solid columns) or PtpA deletion mutant (Striped columns). Counts were transformed to fold-change from matched infection counts in CFU/mL from immediately after infection (T0), (Tfinal/T0). Colonies were grown on 7H10 agar with appropriate supplements for mycobacteria. Data reported as mean ± standard error of the mean (SEM) of at least three technical replicates with n=3. Statistics indicate significance from one-way ANOVA with Dunnett ‘s post-test compared to DMSO. * p < 0.05, ** p < 0.01, *** p < 0.001, **** p < 0.0001.
